# 

**Published:** 1890-06

**Authors:** 


### CONTENTS:

Article I.—The Odontological Society of Great Britain. - 49
" II.—Prosthetic Hygiene. By J. Hall Lewis, D.D.S. - 63
" III.—The Physiology and Treatment of Sensitive dentine. By George M. Keevil. - - - - - 73
" IV.—Aseptic and Antiseptic Dentistry. By H. M. Clifford, D.M.D. - . - - 81

### COMMUNICATIONS.

Post Graduate Dental Association of the United States. - 89
Chloride of Methyl Spray. - - - - - - 90
Chicago Dental Society. - - - - - - 91

### EDITORIAL.

The Law of the State of Virginia Regulating the practice of Dentistry. - - - - - - 92

### MONTHLY SUMMARY.

A Dental operation in which Teeth buried in the Jaw are removed. 95

Our position at the Chair. - - - - - - 95

The Dental Mirror. - - - - - - - - 96

